# Polysaccharide-Based Aerogel Production for Biomedical Applications: A Comparative Review

**DOI:** 10.3390/ma14071631

**Published:** 2021-03-26

**Authors:** Mariangela Guastaferro, Ernesto Reverchon, Lucia Baldino

**Affiliations:** Department of Industrial Engineering, University of Salerno, Via Giovanni Paolo II, 132, 84084 Fisciano, SA, Italy; mguastaferro@unisa.it (M.G.); ereverchon@unisa.it (E.R.)

**Keywords:** agarose, alginate, chitosan, aerogels, supercritical CO_2_, bone regeneration, skin regeneration

## Abstract

A comparative analysis concerning bio-based gels production, to be used for tissue regeneration, has been performed in this review. These gels are generally applied as scaffolds in the biomedical field, thanks to their morphology, low cytotoxicity, and high biocompatibility. Focusing on the time interval 2015–2020, the production of 3D scaffolds of alginate, chitosan and agarose, for skin and bone regeneration, has mainly been investigated. Traditional techniques are critically reviewed to understand their limitations and how supercritical CO_2_-assisted processes could overcome these drawbacks. In particular, even if freeze-drying represents the most widespread drying technique used to produce polysaccharide-based cryogels, supercritical CO_2_-assisted drying effectively allows preservation of the nanoporous aerogel structure and removes the organic solvent used for gel preparation. These characteristics are essential for cell adhesion and proliferation.

## 1. Introduction

In 1931, Kistler [1] produced highly porous materials by removing the liquid part in a gel, working at supercritical conditions. This process avoided shrinkage and solid network deformations during drying, maintaining open porosity and preserving the structure of the gel. In this way, a solid, in which the dispersed phase was air, was obtained, i.e., an aerogel.

In recent years, the research on aerogels production has been widely intensified thanks to the aerogels’ interesting physical and chemical properties [2], such as high porosity (80–99.8%), ultralight weight, thermal resistance (thermal conductivity range from 0.005 to 0.1 W∙mK^−1^), high specific surface area (between 500 and 1200 m^2^∙g^−1^), low dielectric constant (κ value range from 1.0 to 2.0), and low refractive index (≈1.05) [3].

Despite the numerous advantages that can be provided by silica and other inorganic aerogels [4,5,6,7,8], the raw materials of conventional aerogels come from petrochemical-based sources. In response to environmentally friendly requirements, bio/bio-inspired materials have attracted widespread interest thanks to their biocompatibility, biodegradability, and abundance; these are some of the most important requisites for potential biomedical applications, such as tissue engineering (TE), disease diagnosis, drug delivery (DD), and bio-sensing [9,10,11].

The main difference among bio-aerogels, especially polysaccharide aerogels, and other organic (resorcin–formaldehyde, poly(vinyl chloride), and others) or inorganic (silica, alumina, titania, and others) aerogels, is the sequence of steps involved in their preparation. Inorganic aerogel synthesis starts with the hydrolysis and/or polycondensation of alkoxide compounds in the presence of a catalyst [12]; the produced solvogel is formed by a liquid phase embedded in a highly porous solid network. The production of organic aerogels starts with the dissolution of polymers in water or in organic solvents (polysaccharides are generally soluble in aqueous solutions). Then, solution gelation occurs, in which polymer chains rearrange themselves into an open porous network. Gelation can be induced by chemical, enzymatic or physical crosslinking. Chemical cross-linking involves the introduction of permanent linkages by means of a cross-linking agent; for example, glyoxal(dialdehyde), glutaraldehyde (GTA), butane tetracarboxylic acid and citric acid [13,14]. On the other hand, physical cross-linking is characterized by hydrogen bonding, Van der Waals forces, or electrostatic interactions [15,16]. Many polysaccharides can undergo the formation of Van der Waals forces or hydrogen bonding, due to the presence of functional groups localized on their backbones [15].

**Figure 1:**
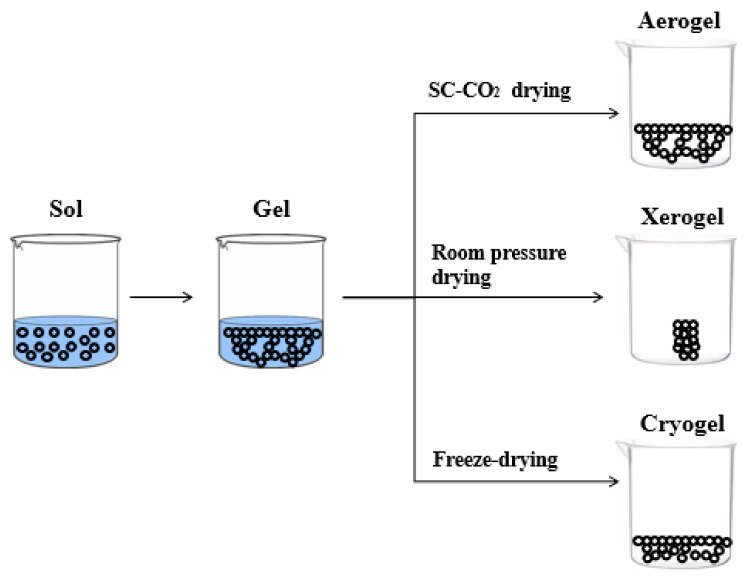
Schematic representation of aerogel, xerogel, and cryogel.

The features of polysaccharide-based aerogels porous structure depend on the drying technique. There are three kinds of solid materials that can be formed after drying, i.e., xerogel, cryogel and aerogel, as shown in Figure 1. The solid material can be termed as xerogel when drying is carried out under ambient pressure and at room temperature, generally, for several days [17,18]. When water (ice) inside the hydrogel is sublimated by freeze-drying, the resultant materials are called cryogels. However, these samples have a macroporous structure, characterized by large and irregular pores [19,20,21,22,23,24].

**Figure 2:**
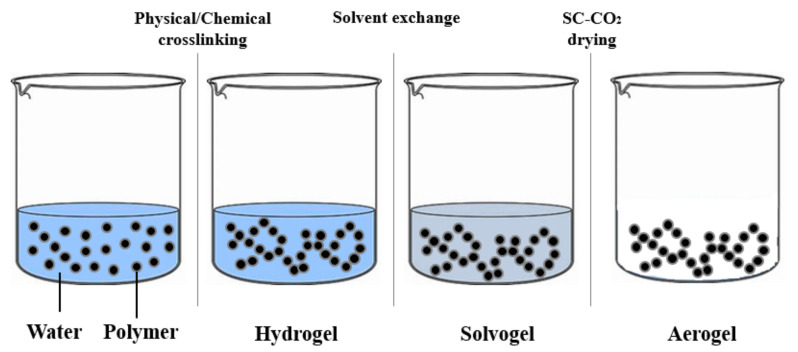
Hydrogel–solvogel–aerogel production.

Aerogels are generally produced by supercritical CO_2_ drying. This technique, whether properly performed by selecting the operative pressure and temperature, avoids the collapse of the native gel nanostructure and preserves the excellent textural properties of the gel [25,26,27,28,29]. Moreover, unlike many organic solvents, supercritical CO_2_ (SC-CO_2_) is non-flammable, inert, non-toxic, has a low cost, and moderate critical values (about 31 °C and 73.8 bar). It is worth mentioning that SC-CO_2_ can form a supercritical mixture with the organic solvent used to prepare the solvogel at mild pressures and temperatures; therefore, during solvent extraction, liquid and gaseous phases become a homogenous phase, avoiding the formation of a liquid–vapor meniscus. In this context, no capillary forces are exerted on pore walls, because the supercritical mixture presents an almost-zero surface tension, and the polymeric matrix does not collapse [27,28,29,30,31,32,33,34]. However, SC-CO_2_ can only be directly used for solvogels, because it shows a limited solubility with water at ordinary pressures and temperatures. For this reason, in the case of hydrogels, water has to be replaced with a suitable solvent miscible in both water and CO_2_, before carrying out the drying process [25] (Figure 2).

Therefore, the aim of this review is to compare the main techniques to produce alginate, chitosan, and agarose aerogels, for skin and bone regeneration. For this purpose, an analysis of the literature, in the time interval 2015–2020, has been performed to highlight advantages and disadvantages of the traditional and innovative techniques investigated.

## 2. Classification

### 2.1. Inorganic Aerogels

Inorganic aerogels are not the main scope of this work; however, they are relevant to understand the possible processes that can be applied to polysaccharide-based aerogels. Much attention has been given to silica aerogels for their use in several industrial applications, including thermal insulation for skylights and windows [4,5]. Silica aerogels have also been used as heat storage devices and as acoustic barrier materials [6]. Thanks to the high porosity and very large surface area, silica aerogels can be also utilized as gas filters, encapsulation media, and hydrogen fuel storage [35].

Silicon alkoxide precursor is reactive enough to form gel networks with other metal oxides [36]; therefore, several studies were carried out to synthesize silica aerogel composites; for example, silica–titania (TiO_2_), silica–carbon and silica–alumina [37,38,39,40,41,42,43,44]. These inorganic aerogels are generally prepared using a two-step sol–gel process, starting from an organic solvent, frequently ethanol. Solvogel preparation involves hydrolysis and alkoxide polycondensation. The obtained solvogel is formed by a solid amorphous structure characterized by an open porosity within the range of 90–95% and nanometric pore diameter. Then, to obtain the corresponding aerogel, the alcoholic solvent is removed from the solid skeleton [45,46]. The main problem during aerogel production is solvent evaporation: when a liquid is evaporated from a fragile and microporous structure, the internal capillary force can lead to a collapse of the pores, with consequent shrinkage of the material. Kistler [1] found a way to obtain monolith silica gels avoiding structure collapse, operating above the solvent critical point, i.e., using the so-called high temperature supercritical drying (HTSCD). In this way, liquid–vapor transition does not occur, and no capillary tension is generated on pore walls. Silica aerogels produced under supercritical conditions are translucent or transparent with low density (30–500 kg/m^3^) and have a considerably high specific surface area (200–1000 m^2^/g). Campbell et al. [47] prepared TiO_2_ aerogels in methanol to increase the surface area of this catalytic material and found that, during supercritical drying, performed at 207 bar, temperatures in the range of 210–300 °C were required for complete solvent removal. At the end of the process, TiO_2_ samples showed a surface area value (200 m^2^/g) three-fold larger than the conventional ones. Kocon et al. [48] produced ultralow density silica aerogels by HTSCD. Solvent composition and solvent quantity were investigated to keep the silica dissolution as low as possible during the supercritical drying; operating in this way, silica aerogels with densities lower than 3 kg/m^3^ were obtained. Moussaoui et al. [49] synthesized TiO_2_ aerogels for photocatalysis applications. In this case, isopropanol was supercritically extracted at 300 °C and 100 bar. A comparison between supercritical drying and ambient pressure drying at 200 °C was performed, and they noted that, under subcritical solvent conditions, the evaporation of the solvent from wet gel led to high stresses in the structure, due to capillary pressure at the gel/solvent interface.

However, HTSCD process may present some problems due to the combination of high temperatures and high pressures, as well as the flammability of the solvents. For this reason, low temperature supercritical drying (LTSCD) has been introduced. During this process, liquid CO_2_ is pumped at a temperature lower than 10 °C, until pressure reaches about 100 bar. When the solvent is completely replaced by CO_2_, temperature is raised up to 40 °C, and the transition of CO_2_ into supercritical conditions is obtained; then, the system is slowly depressurized [50,51,52,53]. Compared to HTSCD, longer operating times are required for this process. Mizushima et al. [50] compared the physical properties of alumina solvogels dried using SC-CO_2_ and ethanol, operating at 80 °C and 157 bar, with alumina solvogels dried under supercritical condition of ethanol (270 °C and 265 bar). Alumina aerogel prepared by supercritical drying had lower densities than alumina xerogel. Moreover, these authors discovered that alumina aerogels were strengthened by treatment performed at higher temperature and pressure. Deng et al. [51] and Kinoshita et al. [52] prepared silica and TiO_2_ nanoaerogels, respectively, using LTSCD. Shimoyama et al. [53] performed SC-CO_2_ drying for the preparation of high-surface area TiO_2_ aerogels from sol–gel routes. It was found that the drying conditions over the critical pressure for the CO_2_ + acetone binary system produced TiO_2_ aerogels characterized by needle-like shape and high specific surface area.

### 2.2. Organic Aerogels

Inorganic aerogels and some organic aerogels are not biocompatible. In order to overcome this drawback, polysaccharide-based aerogels have been widely investigated, frequently starting from water-based solutions. CO_2_ shows a low solubility with water at ordinary process conditions; therefore, it is necessary to exchange it with an intermediate solvent that is miscible both in water and CO_2_, after the hydrogel preparation [54].

Yamashita et al. [55] prepared poly(vinyl chloride) (PVC) aerogels starting from a dimethylformamide (DMF) solution. DMF in the solvogel was firstly exchanged with liquid CO_2_, then supercritically dried at 31 °C and 73.8 bar. Alshrah et al. [56] developed high-porosity resorcinol (RSR)/formaldehyde (FRM) aerogels, through an LTSCD process. After RSR/FRM gelation, the aqueous solution was replaced by acetone. Then, acetone was exchanged with liquid CO_2_ for three days and, at the end, pressure and temperature were increased beyond the CO_2_ critical point. However, the LTSCD process is questionable because CO_2_ exchange is very long and several days are required to obtain a complete solvent removal using liquid CO_2_ [56,57,58].

To overcome this drawback, HTSCD was investigated for organic aerogel manufacturing. Wu et al. [59] prepared an organic aerogel using NaOH-catalyzed polycondensation of RSR/furfural (FRF) and supercritical drying in ethanol, setting pressure at 120 bar and temperature at 255 °C. Chen et al. [60] synthesized lignin/RSR/FRM aerogels using supercritical ethanol drying. Process conditions were set at 250 °C and 100 bar. However, HTSCD cannot be used to produce polysaccharide-based aerogels, because polymeric chains may undergo thermal decomposition. Therefore, different alternative drying techniques have been explored.

The first step in polysaccharide-based aerogel production is the dissolution of the organic compounds in an aqueous solution. Then, the hydrogel is formed by chemical, enzymatic, or physical cross-linking. During the drying process, the liquid surrounding the polymeric network is carefully removed and replaced with air. Drying carried out under ambient pressure for several days can lead to the formation of a condensed structure with an insufficient porosity and large shrinkage; therefore, cryogels produced by freeze-drying have become a common approach. To fabricate cryogels, the gel-like matrices formation occurs in the frozen systems, using physical or chemical gelation. This typically occurs between −5 °C and −20 °C, because most of the solvents crystallize at these conditions. Solvent crystals act as porogens, while the hydrogel constituents remain in liquid micro-phases [21,22,23,24]. Freezing rate can have a relevant impact on the cryogel formation: slower rates may result in larger pores with increased interconnectivity, whereas faster freezing rates produce mechanically weaker cryogels, with a low level of interconnectivity [61]. Mahmoud et al. [62] prepared norfloxacin-loaded scaffolds for wound treatment by freeze-drying, combining collagen (CLG) with two different types of chitosan (CS). These scaffolds were characterized by pores with an average diameter ranging from 150 to 300 µm, which showed an almost-100% drug release within 24 h. You et al. [20] produced metallic nanosilver (NAg)–CLG–CS hybrid scaffolds and investigated their potential effects on wound healing. It was demonstrated that NAg–CLG–CS was bactericidal, anti-inflammatory, and promoted wound healing, regulating fibroblast migration and macrophage activation. Both CLG–CS and NAg–CLG–CS had a mean pore size of 136 ± 5 µm and a porosity of 93.6%. Rubio-Elizalde et al. [21] realized alginate (ALG) cryogels, using polyethylene glycol (PEG)–methyl ether methacrylate (MA) as a plasticizer, with the aim of improving the stability properties of the scaffolds into an aqueous medium. Viability of fibroblasts into the scaffolds was increased with the addition of *Aloe vera* and *Moringa oleifera* extracts. All the obtained scaffolds showed semispherical pores between 50 and 100 μm. In particular, *M. oleifera* significantly increased the scaffold cell proliferation, compared with scaffolds without plant extracts. To mimic the natural structure of tissue extracellular matrix (ECM), Yang et al. [22] prepared a silk fibroin (SF)–hyaluronic acid (HA)–sodium alginate (SALG) composite scaffold by freeze-drying: SF materials are beneficial for fibroblast proliferation, and HA materials are beneficial for cell adhesion. The average pore size (≈93 μm), the mean porosity (≈92%) and the swelling ratio (≈42%) were suitable for fibroblast infiltration and for skin regeneration (SR). Gupta et al. [23] fabricated porous scaffolds for bone regeneration (BR), blending keratin (KRT) and ALG. The solution was kept in a freezer at −20 °C for three days. After that, solvent extraction was performed to obtain a porous scaffold. The morphology of the KRT/ALG scaffold indicated the presence of closed pores having a polydisperse distribution, ranging from 10 to 200 μm. Cheng et al. [24] prepared ALG-based aerogels by ionotropic gelation and freeze-drying, using N,N′-methylenebisacrylamide and carboxy-methylcellulose as reinforcing agents. The final aerogels were characterized by an irregular and closed morphology.

However, the native nanoporous hydrogel morphology is not preserved during freeze-drying. In particular, when an aqueous solution is frozen at an extremely low temperature, the rapid formation of ice nuclei leads to a growth of small ice crystals; although, in any case, not of nanometric dimension [61]. This effect can be considered a relevant drawback for medical applications, because mesoporosity has an important impact on implant topography and scaffold bioactivity. Other problems associated with this technique are a low structural stability and generally weak mechanical properties of the fabricated materials; moreover, the 3D structure, similar to the tissue to be substituted, is frequently missing [63,64].

At this point, the question is: “Is there a way to preserve the delicate polysaccharide–hydrogel structure, maintaining the native macro- and nanoporosity?”. An SC-CO_2_ drying technique has been tested to generate nanoporous polysaccharide 3D aerogels [25]. SC-CO_2_ can only be used directly with organic solvents; therefore, a solvent exchange is required for polysaccharides that form 3D networks in aqueous solution. As the second step, SC-CO_2_-assisted drying is performed using pressures and temperatures that exceed the critical values of the solvent mixture (CO_2_ + organic solvent). In this way, the liquid and gaseous phase form a supercritical mixture: no capillary forces are exerted on pore walls and, therefore, there is no collapse of the polymeric matrix. Using SC-CO_2_ processing, Munor-Ruiz et al. [26] developed a highly porous CLG–ALG–graphene oxide (GO)-based aerogel. Scanning electron microscopy (SEM) images showed a porous interconnected network covered by a nonporous external wall. Baldino et al. [54] produced poly-L-lactid acid (PLLA) aerogels by SC-CO_2_ drying. These structures showed a microporous architecture and, at the same time, maintained the native nanostructure of the gel, consisting of a network of nanofilaments. These scaffolds were also loaded with hydroxyapatite (HAp) nanoparticles to improve the mechanical properties of the PLLA structure. Baldino et al. [27], using the same technique, produced ALG/gelatin (GLT) and CS/GLT [29] aerogels. The results indicated that both ALG/GLT and CS/GLT mixtures formed uniform gels, and SC-CO_2_ drying preserved their delicate nanostructured morphology (≈100 nm), thanks to the near-zero surface tension of the supercritical mixture (CO_2_ + organic solvent) formed during the drying process. Baldino et al. [28] also produced ascorbic acid loaded-SF aerogels by SC-CO_2_ drying. Supercritical assisted process allowed preservation of the SF aerogel morphology at nanoscale, and ascorbic acid release rate was controlled, as well as mechanical properties of the produced aerogels, varying the SF concentration in the starting hydrogel. The same authors [29] prepared CS/GLT aerogels characterized by a Young’s modulus of 181 kPa, larger than that of the single polymers, because a new interpenetrated gel network was obtained.

## 3. Specific Application: Chitosan, Alginate, Agarose Scaffolds for Tissue Engineering

**Figure 3:**
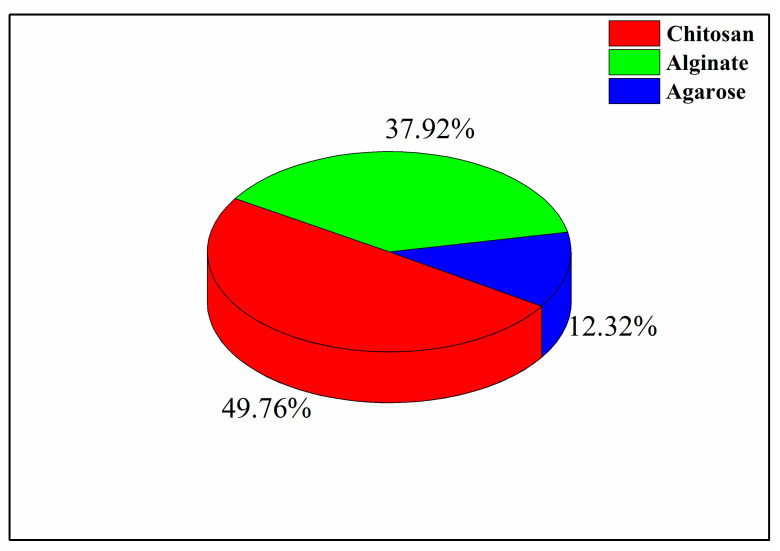
Investigation percentages of chitosan (CS), agarose (ALG) and alginate (AGR) in the literature.

In this review, the main attention will be focused on the production of three polysaccharide-based gels, namely, chitosan (CS), alginate (ALG), and agarose (AGR). These biopolymers have been widely investigated in the literature for tissue engineering applications, as shown in Figure 3. The percentages reported in this figure were calculated using the database Science Direct, looking at the number of papers written in the period 2015–2020, for each biopolymer chosen. The path used was the following: “biopolymer (ALG/CS/AGR), tissue engineering, aerogel/cryogel”.

The following sections will be classified according to the biopolymer and drying technique used, namely, cryogels refers to freeze-drying and aerogels refers to SC-CO_2_ drying.

### 3.1. Skin Regeneration

Skin is the largest organ in the body and protects it against the outside environment and microorganisms. Skin includes the dermis, epidermis, and hypodermis [65]. Wound healing generally involves four stages: (i) hemostasis; (ii) inflammation; (iii) proliferation; and (iv) remodeling.

**Figure 4:**
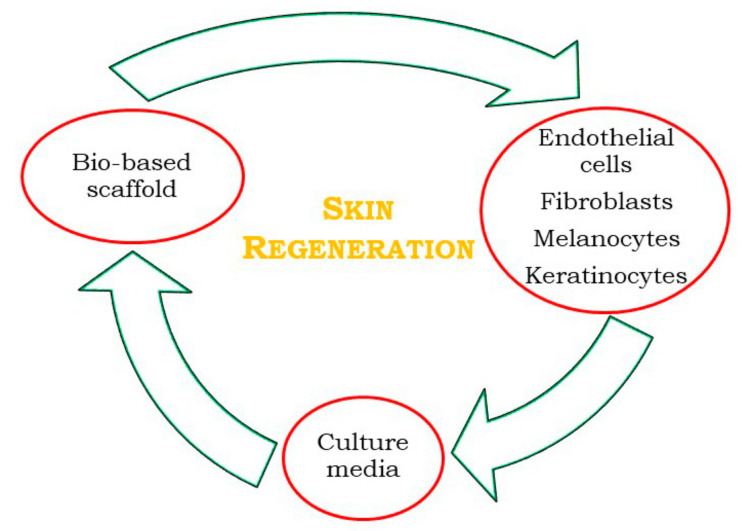
Skin regeneration (SR) process through tissue engineering (TE), adapted from Klar et al. [68].

Polysaccharides have frequently been used in the development of wound dressing materials to improve the efficiency of wound healing (Figure 4), because they can absorb tissue exudates, prevent wound dehydration, and allow oxygen to permeate the wound [66]. Moreover, they can interact with the wound through the release of bioactive molecules, maintaining favorable conditions for the re-establishment of skin integrity and homeostasis. According to the literature, for human skin fibroblast cell growth, pores smaller than 160 μm are required [67], together with a 3D interconnected porous structure, to favor vascularization and cellular colonization [62].

#### 3.1.1. Polysaccharide-Based Cryogels for Skin Regeneration

Chitosan shows antibacterial, hemostatic, and mucoadhesive properties, and can act as a wound healing accelerator [69]. Various studies report that CS promotes the migration of polymorphonuclear neutrophiles (PMNs) and granulation by inducing the proliferation of dermal fibroblasts [70,71,72]. Indeed, during the initial healing, CS favors cell infiltration and migration of neutrophiles and macrophages, decreases tissue scarring, and allows a good re-epithelialization [73]. However, one of the major limitations for CS is its brittleness. Blends with other materials can be an effective way to address this problem. Anjum et al. [73] developed an antimicrobial and scar-preventive hydrogel scaffold to release a model drug, tetracycline hydrochloride (TC), during the SR process. CS, PEG and polyvinyl pyrrolidone (PVP) were blended and subsequently freeze-dried. The presence of CS accelerated wound healing without scar formation, and TC helped in the protection from bacterial invasions. The pore size was in the range of 75–120 μm, depending on the blend composition; PEG addition led to a higher porous structure, and for a CS:PEG (50:50) composition, a porosity near to 70% was observed. Mahmoud et al. [19] produced norfloxacin-loaded CS scaffolds by freeze-drying, for the treatment of wounds. These scaffolds had interconnected pores with an average diameter of 150–300 μm, and the degradation study proved that they could resist rapid degradation upon contact to moisturized surface. Zhu et al. [74] prepared three-dimensional porous flurbiprofen (Flu)-grafted ALG–CS cryogels by freeze-drying combined with amination, for skin TE applications. Compared with the interior of the ALG–CS scaffold, the interior of the ALG–CS–Flu scaffold showed a porous structure with a proper pore size (≈80 µm) that allowed a fast cell in-growth. Moreover, the synergistic effect of the grafted Flu and three-dimensional structure of the scaffold enhanced L929 cell adhesion and proliferation.

Agarose is a polysaccharide with good gelatinizing properties, although it has a low biological activity and a slow degradation rate that limit its role in biomedical engineering. In order to extend its applications to medicine, it is necessary to blend AGR with other bioactive materials for specific functions [75,76,77]. To reach this goal, Ramana Ramya et al. [78] prepared AGR–GLT–HAp composite scaffolds for SR, combining freeze-drying with gamma irradiation at various dosages, i.e., 25 kGy (AGR–GLT–HAp-25), 50 kGy (AGR–GLT–HAp-50) and 100 kGy (AGR–GLT–HAp-100). The scope was to modify the structural and biological properties of the native samples. In particular, at a lower dose (25 kGy), the surface of the samples was characterized by an irregular porous structure which might be due to the localized melting of the polymers.

#### 3.1.2. Polysaccharide-Based Aerogels for Skin Regeneration

Calcium alginate (CAALG) aerogels were produced by Baldino et al. [79] through SC-CO_2_ drying. Franco et al. [80] impregnated CAALG aerogels with mesoglycan (MSG), to favor the re-epithelialization. CAALG was characterized by a nanoporous structure with a mean pore size of about 0.1 μm that was preserved after the MSG impregnation; porosity of CAALG was about 85%. Valchuk et al. [81] prepared aerogels based on CS and ALG for wound healing, using SC-CO_2_ drying. Shrinkage during drying was near to zero and the specific area was 243 m^2^/g, with an average pore size of 0.015 μm. These scaffolds were loaded with an antibiotic, levomycetin. It was estimated that a slower drug release (70% during 5 h) from the aerogel would provide a prolonged and safe DD to the wound surface.

Table 1 reports a summary of the previous papers regarding SR.

### 3.2. Bone Regeneration

**Figure 5:**
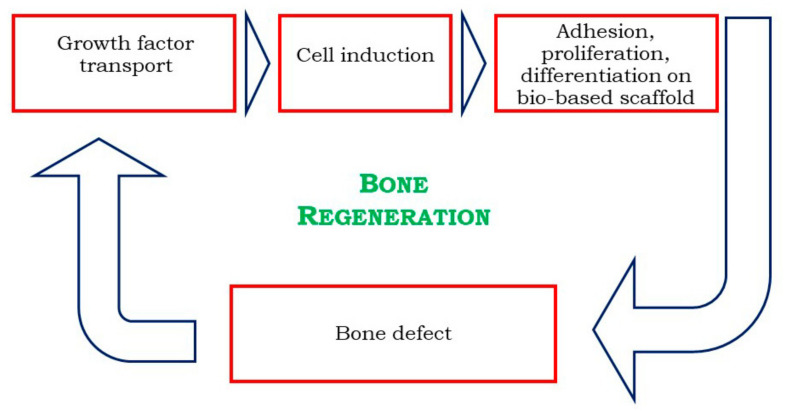
Bone regeneration (BR) through TE, adapted from Bhattacharya et al. [88].

Bone defects are frequently occurring diseases due to tumor, trauma, infection, fracture, and osteoporosis. Traditional treatments mainly include autograft, allograft bone transplantation, prostheses, and artificial bone replacement [82,83]. However, all these methods suffer from some limitations, such as short duration, immune rejection, low biocompatibility, and poor mechanical properties [84]. Therefore, TE has become a promising alternative in bone-related medicine (Figure 5). Scaffolds should have enough mechanical strength because they must provide a support for cell growth, to allow cell vascularization and to mimic the tissue structural environment during in vitro or in vivo study. It was shown that biomaterials possessing large pores (diameter in the range of 100–500 µm) can provide good osteo-integration with the host tissue [63]. Additionally, nanoporosity is as relevant as macroporosity, because it has an impact on implant topography and bioactivity [85]. Nanometric pores are essential for interaction with proteins and cells and can also enhance cell orientation [63,64]. Surface roughness enhances the attachment, proliferation, and differentiation of bone-forming cells, whereas interconnected pores are advantageous over biomaterials containing dead-end pores because a spatial continuous connection of the pore system is decisive for the in-growth of new bone [86]. Finally, a controlled swelling profile is a crucial parameter, because it can affect the structural stability of the scaffolds [87].

#### 3.2.1. Alginate-Based Cryogels for Bone Regeneration

Alginate is widely used for BR thanks to its low cost, low toxicity, and simple gelation mechanism. However, due to its hydrophilic nature, alginate has a reduced capacity to absorb protein that leads to an inhibition of cell adhesion for tissue regeneration [89,90,91,92]. Tohamy et al. [93] and Purohit et al. [94] produced SALG-based nanocomposite scaffolds. The results indicated that the addition of HAp enhanced the scaffold mechanical properties, as well as bioactivity and protein adsorption, whereas the incorporation of GO in the ALG matrix resulted in improved cell attachment and proliferation of MG-63 cells. Afshar et al. [95] used halloysite as a reinforcement for polymeric scaffolds. Some advantages of halloysite (HNT) include hydrophilicity, good dispersion ability, biocompatibility, ability to entrap drugs, and low cost compared to other nanoparticles. The average pore size of CS–ALG was 201.9 ± 16.7 μm, whereas the sizes of CS–ALG–HNT and aminated CS–ALG–HNT corresponded to 183.2 ± 15.2 μm and 181.2 ± 21.1 μm, respectively.

#### 3.2.2. Alginate-Based Aerogels for Bone Regeneration

Baldino et al. [27] realized an interpenetrating polymer network (IPN) of ALG/GLT by SC-CO_2_ drying. Operating at 200 bar and 35 °C, for 8 h, a GTA final residue lower than 3 ppm was obtained. Martins et al. [96] realized macroporous CAALG–starch (STR) aerogels for biomedical applications, trying to obtain pore diameters corresponding to the minimum pore size required for cell migration and penetration into the bulk, as well as for nutrient diffusion into the bulk and waste out of the matrix. Solvogels were treated with pressurized CO_2_ at 50 bar and at room temperature for various time intervals. To introduce macroporosity, the autoclave was depressurized at three rates, 0.1, 10 or 30 bar/min. A significant increase in porosity from 2% up to 25% was achieved by increasing the depressurization rate.

#### 3.2.3. Chitosan-Based Cryogels for Bone Regeneration

Chitosan aerogels can be used as a temporary skeleton, to support and stimulate bone tissue regeneration, while it gradually degrades and is replaced by new bone. They can support cell attachment and proliferation due to their chemical properties; namely, the polysaccharide backbone is structurally similar to glycosaminoglycans, the major component of the ECM of bones [97]. Other advantages of CS scaffolds include the formation of highly porous structures with interconnected pores that ensure good osteoconductivity and good ability to enhance bone formation both in vitro and in vivo. On the other hand, CS is mechanically weak and lacks bioactivity, which limits its use in bone TE. Furthermore, the beginning of acidic by-products degradation in vivo by CS may result in inflammatory reactions. In order to reduce these problems, the integration of calcium phosphates in CS can impart bone-bonding ability and improve mechanical properties. Calcium phosphates can also allow tailoring of the degradation kinetics of the polymer matrix and buffer the acidic resorption by-products of the polymer [98]. Considering that bone ECM is a nanocomposite of minerals and proteins [99], and the major inorganic constituent is represented by the hard and brittle Hap, Tsiourvas et al. [99] produced porous scaffolds based on nano-HAp particles and CS. The mixture was frozen at −25 °C and subsequently freeze-dried. Morphological analysis of scaffolds revealed an open interconnected highly porous structure, with pore dimensions ranging from 200 μm to 700 μm. The mechanical properties of the composite scaffolds containing nano-HAp were significantly higher than CS scaffolds and micron-sized HAp/CS scaffolds, indicating improved dispersion when HAp in the form of nanoparticles was used. Serra et al. [100] produced biocompatible CS/GLT/β-tricalcium phosphate (β-TCP) gels, combining ionic crosslinking using sodium tripolyphosphate (TPP), by freeze-drying. The results revealed that CS/β-TCP, CS/GLT and CS/GLT/β-TCP scaffolds had a compression strength higher than CS scaffolds, whereas a significant decrease in scaffold porosity was observed after the addition of β-TCP.

Cationic amino-groups of CS provide opportunities to form polyelectrolyte complexes (PECs) with anionic materials [101]. Nath et al. [102] immobilized bone morphogenetic protein-2 (BMP-2) in a CS–HA PEC, crosslinked with genipin, for bone TE. After freeze-drying, PECs were porous, with micro- and macropores. A crosslinking of CS–HA PEC scaffolds improved the stability of the scaffolds into aqueous media, depending on the degree of cross-linking. Adhikari et al. [103] combined CS and its oppositely charged derivative, carboxymethyl cellulose (CMC), to form a stable matrix phase, and magnesium gluconate (MgG) was used as dispersed phase. MgG is an organic salt of magnesium that readily dissolves to release Mg^++^ ions that play a crucial role in tissue remodelling and development. These scaffolds were freeze dried for 36 h. SEM analysis showed that the scaffolds had a uniform pore size in the range of 50–150 μm and exhibited no cytotoxicity to osteoblast cells. Liu et al. [104] performed a 3D printing of ALG and CS. Then, the resulted hydrogels were pre-frozen at −20 °C and subsequently freeze dried. Cells adhered to the cryogels and expanded along the direction of the deposited filaments. Shi et al. [105] fabricated biomimetic porous 3D scaffold with a gradient and layered microstructure, by compositing pre-prepared dopamine hydrochloride-modified alginate (ALG-DA) and quaternized chitosan (QCS)-templated HAp by “iterative layering” freeze-drying. The pore sizes of (ALG–DA)–(QCS–HAp0.5), (ALG–DA)–(QCS–HAp1.0), and (ALG–DA)–(QCS–HAp2.0) scaffolds decreased upon the increase in (QCS–HAp) compositions. By combining these three layers into a gradient scaffold, the total porosity was about 72.2 ± 2.3% with a compression module corresponding to 1.7 MPa. Kolanthai et al. [106] also developed a CS-based PEC, using SALG and CS; CLG and GO were added to the polymeric matrix. The chemical crosslinking decreased the pore sizes of the scaffolds to the range of 10–80 μm. Furthermore, the chemical crosslinking of the scaffolds resulted in the loss of CLG fibrous structure, as well as in a gradual decrease in the porous structure and in interconnectivity degree. The swelling ratio of the crosslinked scaffolds was significantly lower when compared to the non-crosslinked scaffolds. Huang et al. [107] added gelatin molecules to thermosensitive chitosan/β-glycerol phosphate disodium salt hydrogels to be applied in the tendon–bone junction, improving its healing capability in a rabbit model.

#### 3.2.4. Chitosan-Based Aerogels for Bone Regeneration

Yilmaz et al. [108] realized biologically safe and mechanically improved three-component aerogels by SC-CO_2_ drying, using GO and HAp as additive materials for CS scaffolds. Indeed, GO is able to overcome some HAp drawbacks, such as low tensile strength, brittleness, and poor impact resistance. Moreover, GO improves cell adhesion, differentiation, and proliferation. Baldino et al. [109] performed a supercritical freeze extraction process (SFEP) for the formation of CS porous structures suitable for BR. The process adopted combined the thermal induced phase separation (TIPS) with SC-CO_2_ drying. Increasing the polymer concentration, the pore size largely decreased, and the scaffold structure became more uniform. It was possible to note that, in addition to the micrometric structure, pore walls were also characterized by a nanofibrous substructure. Ozdemir et al. [110] compared porosity and the osteoblastic cell attachment ability of CS-based scaffolds for TE applications, prepared by two different fabrication techniques, i.e., freeze-drying and SC-CO_2_ drying. Most of the pores were in the range of 20–100 μm for the SC-CO_2_-dried scaffold and 20–200 μm for freeze-dried samples. However, a more homogeneous pore size distribution was achieved using SC-CO_2_ drying. Takeshita et al. [17] performed a comparison between aerogels, xerogels and cryogels. According to SEM observation, CS aerogels consisted of three-dimensionally entangled nanofibers with a diameter of approximately 0.005–0.03 μm. In contrast to the aerogel, no trace of nanofibrous or nanoparticulate structures were observed for xerogels and freeze-dried cryogels that were transparent films with a completely smooth surface at nanoscale. Conzatti et al. [18] produced PECs based on CS and ALG for biomedical applications, using three different drying techniques: hot air-drying, freeze-drying and SC-CO_2_ drying. Xero-samples showed a compact structure, with no external macroporosity, due to the liquid/air surface energy that induced the collapse of PECs polymer chains during drying. The use of the other drying methods prevented the collapse of the polymer chains and the resulting materials showed macroporosities. López-Iglesias et al. [111] proposed a combination of jet cutting and supercritical drying to produce chitosan aerogel microparticles, loaded with vancomycin, for wound healing. These microparticles were characterized by excellent textural properties and provided a sustained antimicrobial activity. Palma et al. [112] found instead that the addition of chitosan scaffolds to blood in dogs did not improve the formation of new mineralized tissues along the root canal walls or histologic evidence of the regeneration of a pulp–dentin complex.

#### 3.2.5. Agarose-Based Cryogels for Bone Regeneration

Some special characteristics of AGR, such as excellent biocompatibility and thermoreversible gelation behavior, support its use in the field of regenerative medicine. This natural polymer, given its similar structure to human ECM, favors cell adhesion, providing adequate permeation of oxygen and nutrients for cell growth [113]. Cell adhesion on AGR substrates can further be improved in stiffness by surface modifications, using nano/microstructures, and through blending with other polymers [114,115]. Luo et al. [116] realized a biomimetic, osteoconductive tricomposite scaffold of N-doped graphene (NG)-HAp hybrids blended with an AGR matrix, via hydrothermal/cross-linking/freeze-drying. The results showed that the introduction of NG and HAp into the aerogel significantly improved its mechanical properties, thereby promoting the proliferation and viability of mesenchymal stem cells (MSCs). AGR also exhibits electro-responsive activity that affects the stability of the structure. Kazimierczak et al. [77] exploited AGR electrophoresis properties, realizing CS–AGR–HAp scaffolds. The scaffolds were produced combining gas-foaming and freeze-drying. The foaming agent, i.e., CO_2_, was produced by the reaction between acetic acid and sodium bicarbonate. The results of absorption demonstrated that both scaffolds exhibited high affinity towards the adsorption of fibronectin and a low one towards albumin. It was also emphasized that the obtained porosity had comparable value to the porosity of trabecular bone that was estimated to be in the range of 50–90%. Sivashankari et al. [117] studied the behavior of porous scaffolds based on AGR and CS with the addition of GO, produced by freeze-drying. Despite the blend AGR–CS showing enhanced swelling behavior and adequate mechanical properties, the composite scaffolds underwent rapid biodegradation. The average pore diameter of AGR–CS scaffolds was found to be 249 ± 35 μm, whereas in the case of AGR–CS–GO composite scaffolds, increasing the GO concentration from 0% to 0.5% w/w, the average pore diameter increased up to 306 ± 46 μm, which then decreased to 237 ± 35 μm, with a further increase in GO. These scaffolds were bio- and hemocompatible, because they showed no apparent cytotoxicity towards the epithelial cells.

Table 2 presents a summary of the papers regarding BR.

## 4. Conclusions

Freeze-drying represents the most widespread drying technique used to produce polysaccharide-based cryogels. However, this process is time-consuming and not particularly promising for TE applications. Indeed, for biomedical applications, scaffolds must simultaneously provide a macroporous structure similar to the tissue to be substituted, and nano-structural characteristics, mimicking the tissue ECM.

These limitations can be overcome by SC-CO_2_ drying. The nanoporous structure, essential for cell adhesion and proliferation, is preserved when this technique is opportunely performed, thanks to the peculiarities of the supercritical mixture CO_2_ + organic solvent formed during drying. In particular, surface tension near to zero avoids the polymeric matrix collapse: the obtained morphology, characterized by nanometer average pore diameters, together with a complete solvent removal, makes these aerogels promising for TE applications.

## Figures and Tables

**Table 1:** Gels applied to skin regeneration.

Authors	Materials	Process	Advantages	Disadvantages
You et al. [20]	NAg particles/CLG/CS	Freeze-drying	Bactericidal properties; Anti-inflammatory properties	Non-uniform morphology can damage fibroblast migration
Rubio-Elizalde et al. [21]	ALG/PEG–MA	Freeze-drying	Delayed degradation kinetics of ALG; Good antioxidant activity; Good antimicrobial activity; Good cell viability	Open pores on the surface can cause device contamination
Mahmoud et al. [62]	Norfloxacin/ CLG/CS	Freeze-drying	Good bio stability; Rapid release of norfloxacin	Polydisperse pores distribution
Anjum et al. [73]	CS/PEG/PVP/TC	Freeze-drying	No-scar formation	Burst effect during drug release
Zhu et al. [74]	Flu/ALG/CS	Freeze-drying + Amidation reaction	Good anti-inflammatory properties and good histocompatibility	Time-consuming process
Ramana Ramya et al. [78]	AGR/GLT/HAp	Freeze-drying + Gamma irradiation	Enhanced hemocompatibility; Enhanced antimicrobial activity and cell viability	Fast dissolution in aqueous medium
Franco et al. [80]	CAALG/MSG	SC-CO_2_ drying + SC-CO_2_ impregnation	Presence of a nanoporous structure; Structure suitable for cell attachment	Energy-consuming process
Valchuk et al. [81]	CS/ALG/ Levomycetin	SC-CO_2_ drying	Nanoporous structure	Burst effect during levomycetin release

**Table 2:** Gels applied to bone regeneration.

Authors	Materials	Process	Advantages	Disadvantages
Takeshita et al. [17]	CS	Air-drying; Freeze-drying; SC-CO_2_ drying	Presence of a suitable nanostructure during SC-CO_2_ drying	Structural degradation during freeze-drying and air-drying; Smooth pores surface for freeze-drying and SC-CO_2_ drying
Conzatti et al. [18]	CS/ALG	Air-drying; Freeze-drying; SC-CO_2_ drying	Mesoporosity was obtained by freeze-drying and SC-CO_2_ drying	Not presence of porosity after air-drying
Gupta and Nayak [23]	KRT/ALG	Freeze-drying	High level of porosity	Time-consuming process; Polydisperse macropores size distribution
Baldino et al. [27]	ALG/GLT	SC-CO_2_ drying	Possible modulation of morphology and mechanical properties of polymeric blends; Presence of nanopores and homogenous structure; Good polymer dispersion	Long time process to completely remove GTA traces
Baldino et al. [54]	CS	SC-CO_2_ drying	Removal of GTA during the process; Presence of nanoporous structure; Good definition of cells three-dimensional scaffolds orientation	Absence of microporosity
Kazimierczak et al. [77]	CS/AGR/HAp	Gas foaming + Freeze-drying	Good value of porosity	Presence of closed pores; Complex process
Baldino et al. [79]	CS/GLT	SC-CO_2_ drying	Possibility to obtain different levels of microporosity and nanoporosity changing polymeric blend compositions; Good mechanical properties	Possible phenomena of separation between the two biopolymers increasing the relative concentrations
Tohamy et al. [93]	SALG/Hydroxyethylcellulose/HAp	Freeze-drying + Cross-linking with Ca^2+^	Improved good mechanical properties	Disomogenous macroporosity and absence of nanoporous structure for cell attachment
Purohit et al. [94]	GO/GLT/ALG	Freeze-drying	Suitable swelling profile	Morphology mainly represented by closed pores
Afshar and Ghaee [95]	CS/ALG/HNT	Freeze-drying + Amination reaction	Homogenous porous structure; Good biochemical characteristics	Complex and time-consuming process
Martins et al. [96]	CAALG/STR	SC-CO_2_ drying using three different depressurization rates	Good biocompatibility; Good bioactivity	Not presence of nanoporous structure
Tsiourvas et al. [99]	Nano-HAp/CS	Freeze-drying	Open interconnected highly porous structure; Improved mechanical properties of CS	Disomogenous macroporosity; HA microparticles were not homogenously dispersed
Serra et al. [100]	CS/GLT/β-TCP	Ionic cross-linking + Freeze-drying	Improved mechanical properties of CS after the addition of GLT and β-TCP; Good level of biomineralization	Drastic decrease in porosity after TCP addition
Nath et al. [102]	BMP-2/CS/HA/Genipin	Freeze-drying	Cross-linking of CS-HA improved the PEC stability in aqueous solution	Burst effect was detected for all samples
Adhikari et al. [103]	CS/CMC/MgG	Freeze-drying	MgG decreased water adsorption of CS scaffolds; Presence of macroporosity and smaller inner pores	Time-consuming process; Burst effect during MgG release
Liu et al. [104]	ALG/CS	Freeze-drying	Good bioactivity of the scaffolds	Samples collapse after processing
Shi et al. [105]	ALG–DA/QCS templated HAp	Iterative layering freeze-drying + Crosslinking by Ca^2+^	Presence of a layered microstructure	Complex process; Uncontrolled degradation profile; Burst effect during levofloxacin release
Kolanthai et al. [106]	SALG/CS/CLG	Freeze-drying	Controlled swelling profile; Improvement of mechanical properties; Good support for cells growth and osteogenic differentiation	The use of a chemical cross-linker resulted in a loss of interconnectivity and in a loss of nanofibrous structure
Yilmaz et al. [108]	CS/GO/HAp	SC-CO_2_ drying	Improved tensile strength of CS scaffolds	Possible residues of GTA; Limitation in GO wt.% used because it can be cytotoxic for living cells; Not presence of nanopores
Baldino et al. [109]	CS	SFEP	Possible modulation of morphology; Uniform structure; High interconnectivity; Presence of micropores and nanopores	Time-consuming process
Ozdemir et al. [110]	CS	Freeze-drying; SC-CO_2_ drying	Smaller and more uniform structure after SC-CO_2_ drying	Not uniform structure during freeze-drying
Luo et al. [116]	NG/HAp/AGR	Hydrothermal + Cross-linking + Freeze-drying	Good mechanical properties	Large pores with irregular shape
Sivashankari and Prabaharan [117]	AGR/CS	Freeze-drying	Good swelling properties; Good mechanical properties; Good bioactivity	The effect of GO on the porosity did not show a precise trend

## Data Availability

Not applicable.

## Abbreviations

AGR, Agarose; ALG, Alginate; ALG–DA, Dopamine hydrochloride-modified alginate; BET, Brunauer–Emmet–Teller; BMP-2, Bone morphogenetic protein-2; BR, Bone regeneration; CAALG, Calcium alginate; CLG, Collagen; CMC, Carboxymethyl cellulose; CS, Chitosan; DD, Drug delivery; DMF, Dimethylformamide; ECM, Extracellular matrix; Flu, Flurbiprofen; FRF, Furfural; FRM, Formaldehyde; GLT, Gelatin; GO, Graphene oxide; GTA, Glutaraldehyde; HA, Hyaluronic acid; HAp, Hydroxyapatite; HNT, Halloysite; HTSCD, High temperature supercritical drying; IPN, Interpenetrating polymer network; KRT, Keratin; LTSCD, Low temperature supercritical drying; MA, Methyl ether methacrylate; MgG, Magnesium gluconate; MSCs, Mesenchymal stem cells; MSG, Mesoglycan; NAg, Nanosilver; NG, N-doped graphene; PBS, Phosphate buffer solution; PEC, Polyelectrolyte complex; PEG, Polyethylene glycol; PLLA, Poly-L-lactid acid; PMNs, Polymorphonuclear neutrophiles; PVC, Poly(vinyl chloride); PVP, Polyvinyl pyrrolidone; QCS, Quaternized chitosan; RSR, Resorcinol; SALG, Sodium alginate; SC-CO_2_, Supercritical CO_2_; SEM, Scanning electron microscopy; SF, Silk fibroin; SFEP, Supercritical freeze extraction process; SR, Skin regeneration; STR, Starch; TC, Tetracycline hydrochloride; β-TCP, β-tricalcium phosphate; TE, Tissue engineering; TiO_2_, Titania; TIPS, Thermal-induced phase separation; TPP, Tripolyphosphate.
